# The authors reply: Letter on: "Myostatin inhibition in combination with antisense oligonucleotide therapy improves outcomes in spinal muscular atrophy" by Zhou *et al*.

**DOI:** 10.1002/jcsm.12608

**Published:** 2020-07-23

**Authors:** Haiyan Zhou, Francesco Muntoni

**Affiliations:** ^1^ Genetics and Genomic Medicine Research and Teaching Department, Great Ormond Street Institute of Child Health University College London London UK; ^2^ NIHR Great Ormond Street Hospital Biomedical Research Centre London UK; ^3^ The Dubowitz Neuromuscular Centre, Developmental Neurosciences Research and Teaching Department, Great Ormond Street Institute of Child Health University College London London UK

**Keywords:** Spinal muscular atrophy, Survival motor neuron protein, Combinatorial therapy

We would like to thank the comments from Dr Chen on our paper on ‘Myostatin inhibition in combination with antisense oligonucleotide therapy improves outcomes in spinal muscular atrophy’.^1^ In this study, we combined the survival motor neuron (SMN)‐restoring antisense oligonucleotide therapy with adeno‐associated virus (AAV)‐mediated myostatin inhibition, and indicated the potential additional therapeutic benefit of the muscle‐enhancing approach in spinal muscular atrophy (SMA) patients treated with nusinersen. We appreciate Dr Chen's comments on our study and the opportunity that this provides us to further expand the increasingly important topic of SMN‐dependent and SMN‐independent approaches for SMA. In our study, we used an AAV‐mediated approach to disrupt myostatin function. This was a preclinical proof‐of‐concept study. We agree that other approaches using recombinant proteins or monoclonal antibodies targeting the myostatin pathway would represent more practical and attractive solutions for clinical applications.

Four SMN‐dependant drugs have been developed for SMA, including antisense drug nusinersen,^2^, ^3^ AAV‐mediated gene therapy Zolgensma,^4^ and the small molecule drugs risdiplam and branaplam.^5^ These SMN‐dependant approaches clearly provide unprecedented clinical improvements for patients with SMA. ,Nevertheless there continues to be an unmet need for SMA patients, which is dependent on the duration of the interval between diagnosis and therapeutic intervention. As SMA is a motor neuron disease, a longer disease duration leads to more severe motor neuron loss and muscle atrophy. SMN‐independent approaches that could complement the previous strategies have therefore an important role especially in chronic symptomatic patients. Various strategies are being developed to target different aspects of SMA pathogenesis. These include neurotransmission enhancer (pyridostigmine bromide, NCT03921528) to improve neuromuscular junction function, which is impaired in SMA patients^6^; a neuroprotection agent to protect motor neurons (olesoxime, NCT01302600 and NCT02628743), which however failed to reach the primary endpoint^7^; fast skeletal muscle troponin activator to slow the rate of calcium release and enhance muscle contraction (CK‐2127107, NCT02644668); and myostatin inhibitor to enhance muscle strength (SRK‐015, NCT02941328). The results of SRK‐015 and CK‐2127107 are expected later on this year from the currently ongoing trials.

It is worthy to note that so far all these clinical studies are designed to test the drug as an independent approach, without the combination with any of the SMN‐dependent treatment. While this is understandable in the early phase trials, it is anticipated that combinatorial therapies with SMN‐restoring drugs would provide the maximal benefit. Future clinical trials on drugs combining both SMN‐dependent and SMN‐independent approaches are hence imperative to further improve the treatment for SMA.

## Author contributions

F. M. and H. Z. discussed, wrote, and approved the correspondence.

## Conflict of Interest

Professor Francesco Muntoni has served on the scientific advisory boards for Sarepta, Pfizer, Roche Biogen, and Avexis; receives research support from Biogen; and has received funding for trials from Sarepta, Avexis, Biogen, PTC, Wave, Roche, and Sarepta Therapeutics. Dr Haiyan Zhou declares no conflicts of interest in this study.

## Funding

This work was supported by the Wellcome Trust (Reference 204841/Z/16/Z), the SMA‐Europe and UK SMA Trust, and NIHR Biomedical Research Centre at UCL Great Ormond Street Hospital.
